# Adaptation and feasibility assessment of an intervention using mHealth and care navigation systems for people living with demencia and their caregivers in Peru

**DOI:** 10.1002/alz.093069

**Published:** 2025-01-09

**Authors:** Miriam Lucar Flores, María Sofía Cuba, Francisco Diez Canseco, Graham Moore, Christopher Butler, Impact Dementia Study Group

**Affiliations:** ^1^ CRONICAS, Universidad Peruana Cayetano Heredia, Lima, Lima Peru; ^2^ Universidad Peruana Cayetano Heredia, Lima, Lima Peru; ^3^ Cardiff University, Cardiff, Wales United Kingdom; ^4^ Imperial College London, London United Kingdom

## Abstract

**Background:**

Dementia is a complex health condition that poses challenges not only to people living with dementia (PLWD) but to their families, the health system and society as a whole. Even though there is still no cure for dementia, different interventions are showing substantial contribution. mHealth‐based assistive technology has shown the potential to provide efficient healthcare for PLWD and their caregivers in cognitive training, health and safety monitoring, educational support, and socialization. Also, Care Navigation Systems offers person‐centered, strengths‐based, and continuous support to PLWD and their caregivers across the illness trajectory and different settings through collaborative problem‐solving and coaching. The IMPACT project will propose innovative solutions for dementia using a collaborative and multidisciplinary approach. For that reason, one of its components aims to adapt an existing intervention to improve the quality of life for PLWD and wellbeing of their caregivers to develop a context‐fit intervention in Peru.

**Methods:**

To adapt an existing intervention from a High‐Income Country to the Peruvian context: The original intervention, The Care Ecosystem, is a supportive care navigation system delivered over the phone and the internet by care team navigators who respond to PLWD and caregivers’ immediate needs, provide personalized support and education using care plan protocols.

*Phase* 1: Will include the revision of the care protocols used by the original intervention to contrast their relevance and cultural fit with the experiences and needs of PLWD and ther caregivers from 4 regions of Peru (Lima, Tumbes, Iquitos, and Huancayo). This process will include a co‐design process with dementia experts, health providers and target population.

*Phase* 2: Conduct a feasibility trial of the adapted intervention on 120 PLWD/caregiver dyas (60 control group, 60 intervention group) during a period of 9 months.

**Result:**

We expect to identify core change mechanisims of the Care Ecosystem program and develop an adapted intervention that can be cosidered feasible to implement within the Peruvian context.

**Conclusion:**

The adaptation of the intervention will offer insights about the convenience of working with evidence‐ based interventions and the opportunities that can arise when they are carefully tailored to different context, norms and existing resources.

